# Role of Glucagon-Like Peptide-1 (GLP-1) Receptor Agonists in Cardiovascular Risk Management in Patients With Type 2 Diabetes Mellitus: A Systematic Review

**DOI:** 10.7759/cureus.45487

**Published:** 2023-09-18

**Authors:** Panah Parab, Priti Chaudhary, Sonia Mukhtar, Ali Moradi, Athri Kodali, Chiugo Okoye, Dhadon Klein, Iman Mohamoud, Olawale O Olanisa, Pousette Hamid

**Affiliations:** 1 Internal Medicine, California Institute of Behavioral Neurosciences & Psychology, Fairfield, USA; 2 Medicine, California Institute of Behavioral Neurosciences & Psychology, Fairfield, USA; 3 Neurology, California Institute of Behavioral Neurosciences & Psychology, Fairfield, USA

**Keywords:** cardiovascular adverse events, glp-1-gip co-agonist, cardiovascular outcomes, diabetes mellitus type 2, glp-1 receptor agonists

## Abstract

People with type 2 diabetes mellitus have a greater risk of developing cardiovascular problems. Since cardiovascular diseases are a major cause of mortality all over the world, we need to find more efficient measures to control this risk in the diabetes population in addition to conventional glycemic control. In this systematic review, we aim to explore the latest findings on the cardiovascular effects of glucagon-like peptide-1 (GLP-1) agonists and dual GLP-1/glucose-dependent insulinotropic peptide (GIP) agonists in patients with type 2 diabetes mellitus. We conducted a comprehensive literature search using PubMed and Google Scholar as the main sources for data collection. We followed the Preferred Reporting Items for Systematic Reviews and Meta-Analyses (PRISMA) 2020 recommendations for conducting this review. The outcomes of interest included mortality due to cardiovascular causes, non-fatal myocardial infarction, stroke, effects on cardiovascular risk factors, heart failure, and development of arrhythmias. After thorough literature screening and quality analysis, 14 articles were finally included for qualitative synthesis. GLP-1 receptor agonists appeared to be effective in reducing the risk of cardiovascular mortality, myocardial infarction, and stroke. They were found to reduce the risk of composite major adverse cardiovascular event (MACE) outcomes by 12-14% when compared to placebo. Their role in preventing heart failure and arrhythmias is uncertain, and further trials are needed to confirm the same. The cardiovascular outcomes of GLP-1/GIP dual agonists are currently under investigation. Studies completed to date show that they do not increase the risk of cardiovascular disease when compared to placebo.

## Introduction and background

Cardiovascular diseases (CVDs) are a leading cause of mortality worldwide [1]. Ischemic heart disease (IHD) and stroke were the top two causes of disability in people over 50 in 2019 and the two leading causes of disease burden in adults between the ages of 25 and 49 [1]. In the United States alone, about 697,000 people died from heart disease in the year 2020, which accounts for one in every five deaths [2,3]. Diabetes mellitus (DM) is an important risk factor for CVD. A large study published in the European Journal of Preventive Cardiology found that the prevalence of diabetes in patients with coronary heart disease was about 30% compared to 10.5% in the general population [4,5]. Unlike the microvascular complications of diabetes, glycemic control alone does not reduce the risk of mortality due to cardiovascular problems [6]. Hence, it is imperative that we find more ways to reduce this risk in the diabetes population.

Glucagon-like peptide-1 receptor agonists (GLP-1 RAs) were initially developed as antihyperglycemic agents and have shown positive results in large-scale cardiovascular outcome trials (CVOTs). They primarily act by inducing glucose-dependent insulin secretion from pancreatic β cells, inhibiting glucagon release from pancreatic α cells, and delaying gastric emptying. These actions lead to a reduction in blood glucose and improved postprandial glucose metabolism. GLP-1 agonists are also known to induce satiety and weight loss through their action on hypothalamic neurons. As a result, liraglutide and semaglutide have been approved for weight management in people with obesity or overweight [7]. Another drug class recently grabbing attention is the dual GLP-1/glucose-dependent insulinotropic polypeptide (GIP) agonists. Tirzepatide (a new dual GLP-1/GIP agonist) displayed better and clinically relevant hemoglobin A1c (HbA1c) reduction when compared to insulin glargine in persons with type 2 diabetes (T2D) and increased cardiovascular risk, with a reduced incidence of hypoglycemia [8].

In December 2008, the US FDA issued a notice mandating manufacturers to conduct additional studies on the effect of drugs used for type 2 DM (T2DM) on atherosclerotic cardiovascular risk [9]. The FDA implemented this requirement in response to a suspected cardiovascular safety concern regarding rosiglitazone, a concern that was later dismissed [10]. Since that time, several studies have been completed across all medication classes, with most of them showing no concern for elevated atherosclerotic cardiovascular risk. Surprisingly, some of these studies have provided evidence for a reduction in major adverse cardiovascular events (MACEs) in addition to the other benefits [11].

In this systematic review, we aim to explore and aggregate the latest literature findings on the cardiovascular effects of GLP-1 agonists and dual GLP-1/GIP agonists in T2DM with or without pre-existing cardiovascular risk factors. Our outcomes of interest included mortality due to cardiovascular causes, non-fatal myocardial infarction (MI), stroke, effects on cardiovascular risk factors, heart failure (HF), and development of arrhythmias. We included clinical trials, meta-analyses, and traditional and systematic reviews that were published in the English language from 2018 to 2023.

## Review

Methods

This systematic review was conducted in accordance with the Preferred Reporting Items for Systematic Reviews and Meta-Analyses (PRISMA) 2020 recommendations [12].

Database and Search Strategy

We conducted our data search from April 16, 2023 to April 23, 2023. We used PubMed and Google Scholar libraries as sources for data collection. We looked for studies related to the cardiovascular effects of GLP-1 agonists and dual GIP/GLP-1 RAs in patients with T2DM. Our detailed search strategy, keywords, and Medical Subject Headings (MeSH) terms used are elaborated in Table 1. Additionally, we also looked for relevant articles in the reference section of selected papers.

**Table 1:** Search strategy. GLP: glucagon-like peptide-1; GIP: glucose-dependent insulinotropic polypeptide; RCT: randomized controlled trial.

Database	Search strategy	No. of records before applying filters	Filters applied	No. of records after applying filters
PubMed	((GLP 1 agonists OR semaglutide OR dulaglutide OR exenatide OR liraglutide OR lixisenatide OR (("Glucagon-Like Peptide 1/adverse effects"[Majr] OR "Glucagon-Like Peptide 1/agonists"[Majr] OR "Glucagon-Like Peptide 1/analogs and derivatives"[Majr] OR "Glucagon-Like Peptide 1/drug effects"[Majr] OR "Glucagon-Like Peptide 1/pharmacology"[Majr] OR "Glucagon-Like Peptide 1/therapeutic use"[Majr])) OR ("Glucagon-Like Peptide 1/adverse effects"[Mesh:NoExp] OR "Glucagon-Like Peptide 1/agonists"[Mesh:NoExp] OR "Glucagon-Like Peptide 1/analogs and derivatives"[Mesh:NoExp] OR "Glucagon-Like Peptide 1/drug effects"[Mesh:NoExp] OR "Glucagon-Like Peptide 1/pharmacology"[Mesh:NoExp] OR "Glucagon-Like Peptide 1/therapeutic use"[Mesh:NoExp])) AND (Tirzepatide OR glucose-dependent insulinotropic polypeptide/glucagon-like peptide 1 (GLP-1) receptor agonist OR Twincretin OR dual glucagon-like peptide-1 (GLP-1) and glucose-dependent insulinotropic peptide (GIP) receptor agonist OR (("Gastric Inhibitory Polypeptide/adverse effects"[Majr] OR "Gastric Inhibitory Polypeptide/agonists"[Majr] OR "Gastric Inhibitory Polypeptide/analogs and derivatives"[Majr] OR "Gastric Inhibitory Polypeptide/drug effects"[Majr] OR "Gastric Inhibitory Polypeptide/pharmacology"[Majr] OR "Gastric Inhibitory Polypeptide/therapeutic use"[Majr])) OR ("Gastric Inhibitory Polypeptide/adverse effects"[Mesh:NoExp] OR "Gastric Inhibitory Polypeptide/agonists"[Mesh:NoExp] OR "Gastric Inhibitory Polypeptide/analogs and derivatives"[Mesh:NoExp] OR "Gastric Inhibitory Polypeptide/drug effects"[Mesh:NoExp] OR "Gastric Inhibitory Polypeptide/pharmacology"[Mesh:NoExp] OR "Gastric Inhibitory Polypeptide/therapeutic use"[Mesh:NoExp])) AND (Diabetes Mellitus OR Diabetes OR (("Diabetes Mellitus, Type 2/drug therapy"[Majr] OR "Diabetes Mellitus, Type 2/therapy"[Majr])) OR ("Diabetes Mellitus, Type 2/drug therapy"[Mesh:NoExp] OR "Diabetes Mellitus, Type 2/therapy"[Mesh:NoExp])) AND (Cardiovascular outcomes OR Cardiovascular events OR Major Cardiovascular adverse events OR Cardiac OR Cardiovascular OR MACE OR (("Cardiovascular Diseases/drug therapy"[Majr] OR "Cardiovascular Diseases/prevention and control"[Majr] OR "Cardiovascular Diseases/therapy"[Majr])) OR ("Cardiovascular Diseases/drug therapy"[Mesh:NoExp] OR "Cardiovascular Diseases/prevention and control"[Mesh:NoExp] OR "Cardiovascular Diseases/therapy"[Mesh:NoExp]))) OR ((GLP 1 agonists OR semaglutide OR dulaglutide OR exenatide OR liraglutide OR lixisenatide) AND (Diabetes Mellitus OR Diabetes) AND (Cardiovascular outcomes OR Cardiovascular events OR Major Cardiovascular adverse events OR Cardiac OR Cardiovascular OR MACE)) OR ((Tirzepatide OR glucose-dependent insulinotropic polypeptide/glucagon-like peptide 1 (GLP-1) receptor agonist OR Twincretin OR dual glucagon-like peptide-1 (GLP-1) and glucose-dependent insulinotropic peptide (GIP) receptor agonist) AND (Diabetes Mellitus OR Diabetes) AND (Cardiovascular outcomes OR Cardiovascular events OR Major Cardiovascular adverse events OR Cardiac OR Cardiovascular OR MACE))	3251	Free full text, clinical trial, meta-analysis, RCT, review, systematic review, English, 2018-2023	583
Google Scholar	(GLP 1 agonists OR dual GLP 1/GIP agonist OR (GLP 1 agonists AND dual GLP 1/GIP agonist)) AND diabetes mellitus type 2 AND (cardiovascular effects OR cardiovascular outcomes)	1310	2018-2023, English	760

Inclusion Criteria

We included peer-reviewed articles and studies published in the English language from 2018 to 2023. The types of studies chosen were clinical trials, meta-analyses, and traditional and systematic reviews. Only the articles available as free full texts were considered.

Exclusion Criteria

We excluded gray literature, studies conducted before 2018, and those published in languages other than English. We also excluded any studies on children, adolescents, animals, and patients with type 1 DM.

Data Extraction and Quality Assessment

After removing duplicate articles from the search, two authors independently conducted screening and data extraction based on inclusion and exclusion criteria. Any disagreements were resolved by consensus, and the opinion of a third reviewer was sought when required. The tools used for critical appraisal were the Cochrane Risk of Bias tool for randomized controlled trials (RCTs) [13], the New Castle Ottawa scale for observational studies [14], the AMSTAR (A MeaSurement Tool to Assess systematic Reviews) checklist for systematic reviews and meta-analysis [15], and the SANRA (Scale for the Assessment of Narrative Review Articles) checklist for narrative reviews [16]. We excluded studies that were of low quality.

Results

Search Results

**Figure 1:**
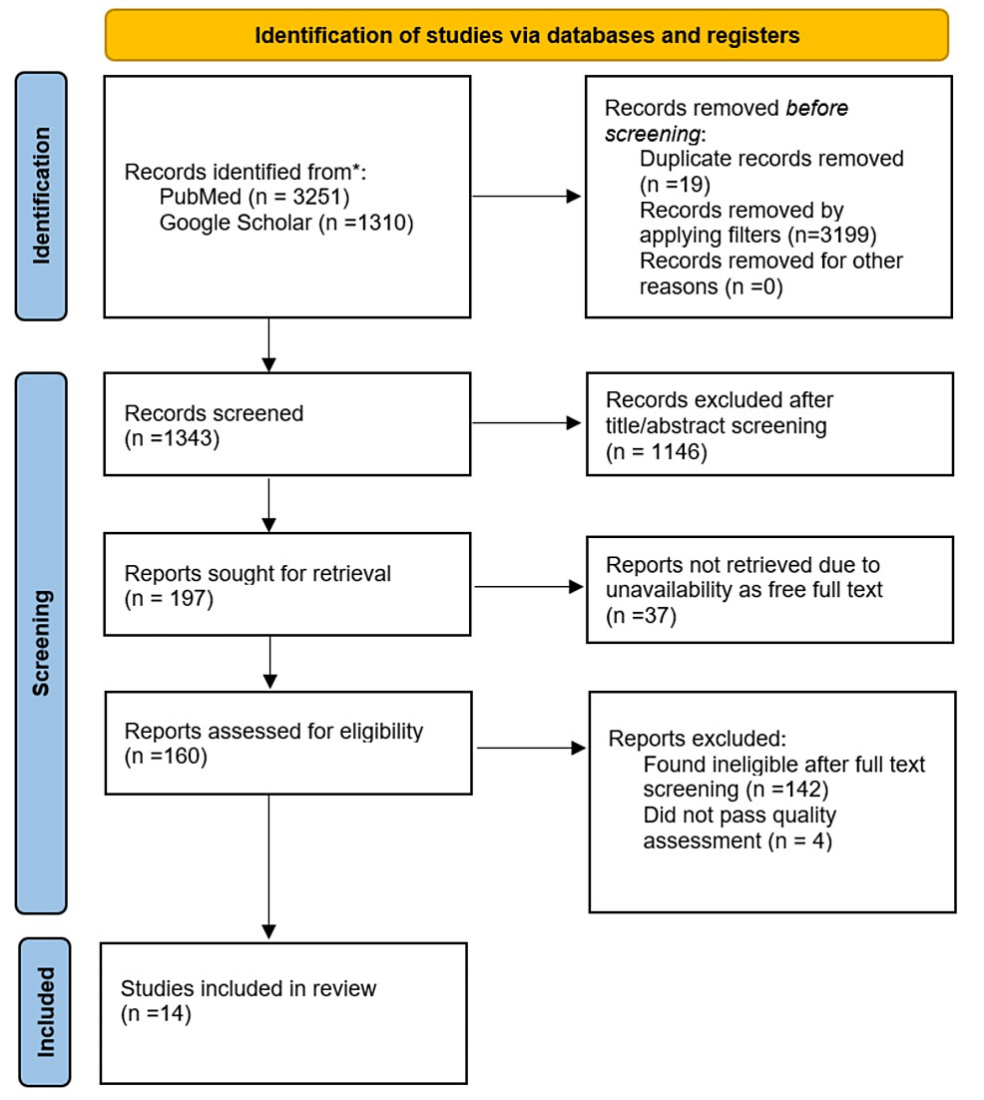
PRISMA flow diagram. PRISMA: Preferred Reporting Items for Systematic Reviews and Meta-Analyses.

Our literature search yielded a total of 4561 articles through PubMed and Google Scholar. Upon applying filters and removing duplicate studies, 1343 articles were retrieved for title and abstract screening. Two individual reviewers conducted the screening independently and excluded 1146 articles for being irrelevant. A similar method of independent screening by two reviewers was used to read the full text, which led to the exclusion of 142 articles. Finally, 18 articles were selected for critical appraisal, of which 14 passed our assessment and were included in the study. Figure 1 shows the detailed PRISMA flow diagram [12].

Critical Appraisal

We assessed the quality of our selected papers using quality appraisal tools. We used the AMSTAR (a measurement tool to assess the methodological quality of systematic reviews) for systematic reviews and meta-analyses and the SANRA (Scale for the Assessment of Narrative Review Articles) for traditional reviews. The detailed quality assessment of the included studies is shown in Table 2. Only those studies that scored above 75% were finally included.

**Table 2:** Critical appraisal of included studies. AMSTAR: A MeaSurement Tool to Assess systematic Reviews.

Title	Author	Type of study	Quality assessment tool used	Quality score
The longer-term benefits and harms of glucagon-like peptide-1 receptor agonists: a systematic review and meta-analysis	Alexander et al. [17]	Systematic review and meta-analysis	AMSTAR	91%
Heterogeneity of antidiabetic treatment effect on the risk of major adverse cardiovascular events in type 2 diabetes: a systematic review and meta‑analysis	D’Andrea et al. [18]	Systematic review and meta-analysis	AMSTAR	100%
GLP‑1 receptor agonists and cardiorenal outcomes in type 2 diabetes: an updated meta‑analysis of eight CVOTs	Giugliano et al. [19]	Meta-analysis	AMSTAR	91%
The effect of DPP‑4 inhibitors, GLP‑1 receptor agonists and SGLT‑2 inhibitors on cardiorenal outcomes: a network meta‑analysis of 23 CVOTs	Giugliano et al. [20]	Meta-analysis	AMSTAR	91%
Dipeptidyl peptidase-4 inhibitors, glucagon-like peptide 1 receptor agonists and sodium-glucose co-transporter-2 inhibitors for people with cardiovascular disease: a network meta-analysis	Kanie et al. [21]	Meta-analysis	AMSTAR	100%
Effects of GLP-1 receptor agonists on cardiovascular outcomes in patients with type 2 diabetes and chronic kidney disease: a systematic review and meta-analysis	Kelly et al. [22]	Systematic review and meta-analysis	AMSTAR	82%
A meta-analysis evaluating indirectly GLP-1 receptor agonists and arrhythmias in patients with type 2 diabetes and myocardial infarction	Liu et al. [23]	Meta-analysis	AMSTAR	82%
Glucagon‑like peptide‑1 (GLP‑1) receptor agonists and cardiovascular events in patients with type 2 diabetes mellitus: a meta‑analysis of double‑blind, randomized, placebo‑controlled clinical trials	Qin et al. [24]	Meta-analysis	AMSTAR	100%
Tirzepatide cardiovascular event risk assessment: a pre-specified meta-analysis	Sattar et al. [25]	Meta-analysis	AMSTAR	82%
Effects of GLP-1 receptor agonists on arrhythmias and its subtypes in patients with type 2 diabetes: a systematic review and meta-analysis	Wei et al. [26]	Systematic review and meta-analysis	AMSTAR	91%
Risk of stroke and retinopathy during GLP-1 receptor agonist cardiovascular outcome trials: an eight RCTs meta-analysis	Wei et al. [27]	Meta-analysis	AMSTAR	82%
Cardiovascular and renal outcomes with SGLT‑2 inhibitors versus GLP‑1 receptor agonists in patients with type 2 diabetes mellitus and chronic kidney disease: a systematic review and network meta‑analysis	Yamada et al. [28]	Systematic review and meta-analysis	AMSTAR	91%
The potential of glucagon-like peptide-1 receptor agonists in heart failure	Kreiner et al. [29]	Narrative review	SANRA	92%
GLP-1 receptor agonists for the reduction of atherosclerotic cardiovascular risk in patients with type 2 diabetes	Marx et al. [30]	Narrative review	SANRA	92%

Study Characteristics

At the end of the screening and quality check, 14 articles were finally included for review. Out of the total, 12 were meta-analyses and two were traditional reviews. Table 3 shows the study characteristics in detail.

**Table 3:** Characteristics of included studies. MACEs: major adverse cardiovascular events; GLP-1 RA: glucagon-like peptide-1 receptor agonists; CVD: cardiovascular disease; CVOTs: cardiovascular outcome trials; SGLT-2: sodium-glucose cotransporter-2; RCTs: randomized controlled trials; T2DM: type 2 diabetes mellitus; CKD: chronic kidney disease; RR: relative risk; MI: myocardial infarction; eGFR: estimated glomerular filtration rate; HFpEF: heart failure with preserved ejection fraction; ASCVD: atherosclerotic cardiovascular disease​​​​​​​; HbA1c: hemoglobin A1c.

Author	Name of journal and publication year	Type of study	Patient population	Outcome
Alexander et al. [17]	Journal of General Internal Medicine, 2022	Systematic review and meta-analysis	45 trials comprising 71,517 patients were included	The three-component MACE outcome favored GLP-1 RA as compared to placebo (RR: 0.87, 95% CI: 0.82–0.93, I^2^ = 23%). GLP-1 RAs led to fewer strokes (RR: 0.86, 95% CI: 0.78–0.95, I^2^ = 0%). GLP-1 RAs compared to placebo were also associated with significant reductions in cardiovascular risk factors
D’Andrea et al. [18]	Cardiovascular Diabetology, 2020	Systematic review and meta-analysis	10 trials enrolling 89,790 patients were included	GLP-1 RA drugs showed a 12% overall reduction in MACEs (HR: 0.88; 95% CI: 0.82–0.94)
Giugliano et al. [19]	Cardiovascular Diabetology, 2021	Meta-analysis	Eight CVOTs enrolling 60,080 patients were included of which 72.4% had established CVD	GLP-1 RAs reduce the risk of MACE by 14% compared to placebo in patients with T2DM over a period of 1.3-5.4 years (HR: 0.86; 95% CI: 0.79–0.94; P = 0.006)
Giugliano et al. [20]	Cardiovascular Diabetology, 2022	Meta-analysis	23 trials enrolling a total number of 181,143 participants were included	GLP-1 RA can reduce MACE by 13% and the risk of non-fatal stroke compared to placebo; SGLT-2 inhibitors are superior in reducing cardiovascular death, hospitalization for HF
Kanie et al. [21]	Cochrane Database of Systematic Reviews, 2021	Meta-analysis	20 RCTs enrolling 129,465 participants were included in meta-analysis (31 studies were used for qualitative analysis)	GLP-1 RA may lower the risk of CVD mortality and all-cause mortality in patients with established CVD, according to meta-analyses of moderate- to high-certainty evidence; moderate-certainty evidence is probable in favor of using GLP-1 RA to lower fatal and non-fatal stroke
Kelly et al. [22]	Pharmacotherapy, 2022	Systematic review and meta-analysis	Four RCTs comprising 7130 patients with T2DM and CKD were included	In a subset population with T2DM and CKD, GLP-1 RAs were not linked with a lower risk of the composite cardiovascular endpoint (three-point composite MACE), compared to placebo (odds ratio: 0.80; 95% CI: 0.59–1.07; P = 0.13)
Liu et al. [23]	Frontiers in Cardiovascular Medicine, 2022	Meta-analysis	Five RCTs with 31,314 patients, which had at least 30% patients with T2DM and MI, were included	GLP-1 RAs may be linked with reduced risk for atrial arrhythmias (RR: 0.81; 95% CI: 0.70–0.95).; GLP-1 RAs appear to have a stronger anti-atrial arrhythmia impact in patients with T2DM and MI
Qin et al. [24]	BMC Endocrine Disorders, 2022	Meta-analysis	Six RCTs with a total of 52,821 patients were included	GLP-1 RA therapy reduced mortality from cardiovascular causes (RR: 0.90; 95% CI: 0.83–0.97; P = 0.004) and fatal or non-fatal stroke (RR: 0.85; 95% CI: 0.77–0.94; P = 0.001)
Sattar et al. [25]	Nature Medicine, 2022	Meta-analysis	Seven RCTs with a total of 7215 patients	Participants with T2DM who took tirzepatide experienced no increased risk of serious cardiovascular problems across a spectrum of T2DM duration and cardiovascular risk levels (HR: 0.80; 95% CI: 0.57–1.11)
Wei et al. [26]	Frontiers in Endocrinology, 2022	Systematic review and meta-analysis	Eight CVOTs were included with a total of 60,081 participants	GLP-1 RA therapy has no discernible impact on the risk of severe arrhythmias in T2DM patients (RR: 0.96; 95% CI: 0.96–1.05; P = 0.36)
Wei et al [27]	Frontiers in Endocrinology, 2022	Meta-analysis	Eight RCTs with a total patient population of 60081 were included	GLP-1 RA significantly reduces the risk of total stroke (RR: 0.83; 95%CI: 0.73-0.95; P = 0.005), as well as ischemic stroke (RR: 0.83; 95%CI: 0.73-0.95; P = 0.008) in type 2 diabetes with cardiovascular risk factors
Yamada et al. [28]	Cardiovascular Diabetology, 2021	Systematic review and meta-analysis	13 studies were selected with a total of 32,949 patients	GLP-1 RAs did not lead to significantly lower cardiovascular endpoints in patients with T2DM and CKD (eGFR < 60 mL/min/1.73 m^2^) (RR: 0.91; 95% CI: 0.80–1.04)
Kreiner et al. [29]	Frontiers in Physiology, 2022	Narrative review	Eight CVOTs with a total of 60,081 participants and one meta-analysis were included	People with HF who have or are at risk of having obesity-related HFpEF are most likely to benefit from GLP-1 RA therapy
Marx et al. [30]	Circulation, 2022	Narrative review	Eight CVOTs with a total of 60,081 participants were included	Numerous studies have demonstrated that GLP-1 RAs lower cardiovascular risk in individuals with diabetes and ASCVD, or diabetes and high cardiovascular risk without regard to HbA1c

Discussion

In this systematic review, we found that GLP-1 RAs have a positive effect on cardiovascular outcomes in people with T2DM. High certainty evidence indicates a reduction of both cardiovascular mortality and all-cause mortality by GLP-1 RAs when compared with placebo [21].

Effects on MACE

Several RCTs have been conducted to date to study the cardiovascular outcomes of GLP-1 RA. A meta-analysis of these trials conducted by Giugliano et al. found that when compared to a placebo, GLP-1 RA lowers MACE risk in T2DM patients by 14% [19]. The three MACE components, CV mortality (reduced by 13%), non-fatal stroke (reduced by 16%), and non-fatal MI (reduced by 9%, although the level of reduction was not statistically significant), are likewise decreased by GLP-1 RA. This reduction in risk was independent of the chemical makeup of these medications (exendin-4-based or human equivalents). Additionally, Giugliano et al. discovered that these medications decreased the risk of MACE to a greater extent in patients with known cardiovascular illness compared to those without established CVD (16% vs. 6% reduction, respectively). A similar finding was observed by D’Andrea et al., wherein patients with pre-existing CVD showed a 14% reduction of MACE (HR: 0.86; CI: 0.80-0.93) on GLP-1 RA, while those at high risk of CVD, but without a history of cardiovascular events, seemed to have minimal or no effect (HR: 0.94; CI: 0.82-1.07) [18]. In the same analysis, baseline HbA1c level was discovered to be an effect modifier in addition to cardiovascular history. Individuals with baseline HbA1c levels that were equal to or higher than 8% showed trends toward significant decreases in the risk of MACE compared to individuals with baseline HbA1c values below 8%. The trials included in the meta-analysis, however, enrolled a majority of, and occasionally only, patients with established CVD and used inconsistent definitions of established CVD. Therefore, additional research focusing specifically on this population would be required to determine whether or not GLP-1 RAs are effective for the primary prevention of cardiovascular events.

Another study conducted by Alexander et al. focused mainly on the longer-term benefits and harms of GLP-1 RAs on the cardiovascular system. Their results provide reassurance that the reduction in MACE and cardiovascular risk factors associated with GLP-1 RA use as compared to placebo was maintained at the end of one year [17]. GLP-1 RAs have been found to significantly reduce the risk of non-fatal stroke when compared with placebo [17,19,21]. According to a study conducted on the GLP-1 RA CVOTs, these medications reduced the risk of total stroke as well as ischemic stroke by approximately 17% in T2D patients [27]. However, they did not lower the risk for hemorrhagic stroke with statistical significance, although the relative risk (RR) was similar to that for ischemic stroke. Among Asian patients with T2DM who had dyslipidemia or hypertension but no known atherosclerotic cardiovascular illnesses, retrospective cohort research published in 2022 found a connection between longer use and larger dosages of GLP-1 RAs and a reduced risk of hospitalization for ischemic stroke [31]. In a further investigation in 2021 examining the efficacy and safety of sodium/glucose cotransporter-2 inhibitor (SGLT2i), GLP-1 RA, and dipeptidyl peptidase 4 inhibitor (DPP4i), it was discovered that just GLP-1 RAs were connected to a lower chance of stroke in comparison with placebo (RR: 0.85, 95% CI: 0.76-0.94) [32].

Effects on HF

Currently, there is sparse evidence on the effects of GLP-1 RAs on HF. Sattar et al. conducted a meta-analysis of the data available from the CVOTs. According to their research, the GLP-1 RA could considerably lower the incidence of HF hospital admission by 11% (HR vs. placebo of 0.89; 95% CI: 0.82-0.98) [33]. All eight CVOTs for GLP-1 RAs, with the exception of semaglutide (HR: 1.11, 95% CI: 0.77-1.61), show HRs of <1. For this secondary outcome, only efpeglenatide and albiglutide demonstrated statistical significance. This may imply that Sattar et al.'s meta-analysis, which identified a benefit of GLP-1 RAs on HF hospitalization, was influenced by the data from the efpeglenatide and albiglutide CVOTs. Post hoc analyses of the CVOT for efpeglenatide revealed a significant 39% RR reduction in HF requiring hospitalization (HR: 0.61; 95% CI: 0.38-0.98). Even though the CVOTs frequently featured a significant count of people with T2D and HF at enrollment, it must be highlighted that the majority of the CVOTs did not analyze outcomes based on the presence of HF at enrollment. Additionally, the studies' definitions of HF varied and were generally vague [29].

Two studies, LIVE (24-week study in adults with stable chronic HF with or without diabetes) and FIGHT (effects of liraglutide on clinical stability among patients with advanced HF and reduced ejection fraction) [34,35], were conducted to examine the effect of liraglutide on HF. In either experiment, liraglutide was unable to show any statistically significant advantages or disadvantages. As a result, the findings regarding GLP-1 RA's effects on HF are mostly equivocal, and additional studies are required to validate such effects.

Cardiovascular Effects in Patients With CKD

Two of the included studies focused on the cardiovascular outcomes of GLP-1 RAs in patients with T2DM and pre-existing CKD. CKD was defined as a reduced baseline eGFR below 60 mL/min/1.73 m^2^. Neither of the studies observed a statistically significant benefit of GLP-1 RAs in reducing composite cardiovascular outcomes in this subset of patients [22,28]. However, a subsequent analysis among GLP-1 RA subclasses revealed that GLP-1 analogs significantly reduced the risk of MACE, while exendin-4 analogs did not. Hence, the final results of the above two studies appear to be influenced by the neutral cardiovascular effects of exenatide (HR: 0.91; 95% CI: 0.83-1.00) and the fact that exenatide CVOT had the largest sample size among the GLP-1 RA CVOT included in the analysis. However, Kelly et al.'s meta-analysis revealed that other GLP-1 RAs were linked to lower cardiovascular event rates in CKD patients, with liraglutide and once-weekly semaglutide showing the greatest absolute reductions [22]. Natriuresis-induced BP reduction, a decrease in reactive oxygen species and inflammation, and an improvement in endothelial function are some potential pathways for these positive benefits. To confirm these effects of GLP-1 RAs in patients with CKD, additional research is required.

Arrhythmias

Wei et al. conducted a systematic review and meta-analysis of data extracted from eight CVOTs for GLP-1 RA to explore their effects on different arrhythmias. They included 60,081 participants of which 76.7% had a history of cardiovascular disease (CVD). Their research revealed that neither the risk of specific types of arrhythmias nor the risk of all arrhythmias was affected by GLP-1 RA therapy (RR: 0.96, 95% CI: 0.96-1.05, P = 0.36) [26]. The use of GLP-1 RAs, however, may be linked to a reduced risk of atrial arrhythmias, according to Liu et al.'s meta-analysis of five trials including 31,314 patients [23]. Only semaglutide was found to lower the risk of atrial arrhythmias and AF, according to the subgroup analysis; the other GLP-1 RAs showed no anti-arrhythmic effect. To be included in this analysis, however, trials had to have at least 30% of their patients with T2DM and MI. The study's findings thus imply that individuals with T2DM and MI appear to be more responsive to the anti-atrial arrhythmia action of GLP-1 RAs.

Effects on Cardiovascular Risk Factors

A meta-analysis of 45 trials comprising 71,517 patients conducted by Alexander et al. in 2022 observed favorable effects of GLP-1 RAs on cardiovascular risk factors at the end of at least one year. GLP-1 RAs led to lower HbA1c levels compared to placebo (MD = −0.67%; 95% CI: −0.77 to −0.58%; I^2^ = 93%) [17]. Trials where participants received background medications at baseline as well as those where participants did not receive other anti-hyperglycemic medications in addition to the study drug showed a similar reduction in HbA1c. GLP-1 RAs also reduced systolic blood pressure (MD = −1.75 mmHg; 95% CI: −2.14 to −1.35 mmHg; I^2^ = 48%), weight (MD −1.84 kg; 95% CI: −2.37 to −1.30 kg; I^2^ = 95%), BMI (MD = −1.12 kg/m^2^; 95% CI: −1.67 to −0.57 kg/m^2^; I^2^ = 96%), and low-density lipoprotein (LDL) (MD = −0.04 mmol/L; 95% CI: −0.06 to −0.02; I^2^ = 0%) when compared to placebo. On the other hand, heart rate increased by about 2 bpm (MD = 2.22 bpm; 95% CI: 1.69-2.75 bpm; I^2^ = 88%). It must be noted that there was significant heterogeneity among several of the above outcomes. However, subgroup analysis generated consistent results.

Dual GLP-1/GIP Agonists

Tirzepatide is a single modified peptide with GIP and GLP‐1 RA approved for the treatment of people with T2D in the United States. Its cardiovascular outcomes are currently under investigation. The drug is also being investigated for its effects on chronic weight management, heart failure with preserved ejection fraction (HFpEF), obesity, and non‐alcoholic steatohepatitis. In a pre-specified meta-analysis of phase two and phase three trials, Sattar et al. observed that tirzepatide was not associated with increased risk of the MACE-4 (composite of cardiovascular death, MI, stroke, and hospitalization because of unstable angina) outcome (HR: 0.80; 95% CI: 0.57-1.11), cardiovascular death (HR: 0.90; 95% CI: 0.50-1.61), MI (HR: 0.76; 95% CI: 0.45-1.28), stroke (HR: 0.81; 95% CI: 0.39-1.68), and hospitalization for unstable angina (HR: 0.46; 95% CI: 0.15-1.41) [25]. The overall cardiovascular results show that tirzepatide treatment, when compared to placebo or comparators not known to be cardioprotective, is not associated with an increased risk of CVD. This treatment was given for a median of just over a year at a mean randomization dose of 9.9 mg per week to a population in which just over one-third had already developed CVD. In subgroup analyses by sex, age, baseline HbA1c, race, US or non-US clinical sites, or baseline SGLT2i use for the primary MACE-4 outcome, no significant effect modification was discovered. According to studies, tirzepatide was superior to placebo, semaglutide 1 mg/week, dulaglutide 1.5 mg/week, insulin degludec 100 U/mL, and semaglutide or insulin glargine 100 U/mL in lowering HbA1c and weight in T2D patients throughout a 26- to 52-week treatment period [36,37]. Once the tirzepatide CVOT results are released, it will be known if or not this advantage over GLP-1 RA will extend to cardiovascular outcomes.

Strengths and Limitations

The strengths of this systematic review are the inclusion of the most recent studies on cardiovascular outcomes of GLP-1 RAs, the large sample sizes in almost all the studies, and the high quality of trials on which the studies were based. We included only those studies that had a low risk of bias. Limitations include excluding studies conducted before 2018, those published in languages other than English, and articles unavailable as free full text. Our review was mainly based on meta-analyses and narrative reviews of previously conducted RCTs. While this provides a good picture of the overall effects of GLP-1 RA as a class, we may have missed the specific effects of individual drugs belonging to the class.

## Conclusions

In patients with T2DM, GLP-1 RAs are associated with a significant 12-14% reduction in three-point composite MACE outcome consisting of cardiovascular mortality, non-fatal MI, and non-fatal stroke compared to placebo. They also significantly reduce the risk of ischemic stroke in T2D patients with cardiovascular risk factors. These effects appeared to be more pronounced in people with pre-existing CVD. Further studies are required to elucidate the role of GLP-1 RA in HF and cardiac arrhythmias. Tirzepatide, the dual GLP-1/GIP agonist approved for treating T2DM, is being investigated for cardiovascular outcomes. This novel class of drugs needs further exploration to unravel its benefits or harms in the treatment of DM. Additionally, it is unknown whether these novel antihyperglycemic medications have cardioprotective properties irrespective of diabetic status, and further investigation may hold the potential to pave the way to a new era of cardiovascular risk management.
